# Ultrahigh‐Power Pseudocapacitors Based on Ordered Porous Heterostructures of Electron‐Correlated Oxides

**DOI:** 10.1002/advs.201500319

**Published:** 2016-01-22

**Authors:** Xing‐You Lang, Bo‐Tian Liu, Xiang‐Mei Shi, Ying‐Qi Li, Zi Wen, Qing Jiang

**Affiliations:** ^1^ Key Laboratory of Automobile Materials Jilin UniversityMinistry of Educationand School of Materials Science and EngineeringJilin UniversityChangchun130022P.R. China

**Keywords:** electrochemical energy storage, electron‐correlated oxides, porous electrodes, pseudocapacitors

## Abstract

Nanostructured transition‐metal oxides can store high‐density energy in fast surface redox reactions, but their poor conductivity causes remarkable reductions in the energy storage of most pseudocapacitors at high power delivery (fast charge/discharge rates). Here it is shown that electron‐correlated oxide hybrid electrodes made of nanocrystalline vanadium sesquioxide and manganese dioxide with 3D and bicontinuous nanoporous architecture (NP V_2_O_3_/MnO_2_) have enhanced conductivity because of metallization of electron‐correlated V_2_O_3_ skeleton via insulator‐to‐metal transition. The conductive V_2_O_3_ skeleton at ambient temperature enables fast electron and ion transports in the entire electrode and facilitates charge transfer at abundant V_2_O_3_/MnO_2_ interface. These merits significantly improve the pseudocapacitive behavior and rate capability of the constituent MnO_2_. Symmetric pseudocapacitors assembled with binder‐free NP V_2_O_3_/MnO_2_ electrodes deliver ultrahigh electrical powers (up to ≈422 W cm^23^) while maintaining the high volumetric energy of thin‐film lithium battery with excellent stability.

## 1 Introduction

This is an open access article under the terms of the Creative Commons Attribution License, which permits use, distribution and reproduction in any medium, provided the original work is properly cited.

With fast‐growing demands for energy storage devices that can store/deliver high‐density energy at rapid charge/discharge rates,^1^, ^2^ enormous research interest has recently been stimulated in exploring pseudocapacitive materials as electrodes in electrochemical capacitors for achieving much higher levels of energy storage than carbon electrode materials.^1^, ^3^, ^4^, ^5^, ^6^, ^7^ Unlike electrochemical double‐layer capacitors (EDLCs),^8^, ^9^, ^10^, ^11^, ^12^ in which charge storage is achieved by nonfaradaic electrostatic adsorption in nanostructured carbons with low intrinsic capacitance (≈20 μF cm^−2^_carbon_),^1^, ^13^, ^14^ pseudocapacitors store high‐density energy on pseudocapacitive materials by fast and reversible surface redox reactions at or near the electrode/electrolyte interface.^1^, ^2^, ^3^, ^4^, ^5^, ^6^, ^7^, ^15^, ^16^, ^17^ The surface mechanisms are fundamentally distinguished from rate‐limited volumetric reactions in batteries by short charge/discharge time, high power density and long‐term cycling stability.^1^, ^2^, ^3^, ^18^ These advantageous features enlist pseudocapacitors to be attractive alternatives or complements to batteries for many high‐power applications in hybrid electric vehicles, portable electronic devices and renewable energy.^1^, ^2^, ^3^, ^4^, ^8^, ^9^, ^13^ However, conventional pseudocapacitors made from state‐of‐the‐art electrode materials, typically transition‐metal oxides (TMOs) such as MnO_2_,^5^, ^15^, ^19^, ^20^ TiO_2_,^6^, ^16^ and Co_3_O_4_,^19^, ^21^ often exhibit much lower power capability than EDLCs due to their intrinsically poor conductivity.^15^, ^21^ It thus remains a primarily challenge in realizing high‐power and high‐energy densities in pseudocapacitors, which requires pseudocapacitive electrode materials simultaneously providing large specific surface area and ultrahigh transports of ions and electrons.^22^, ^23^, ^24^ In this regard, controlling nanostructures and exploring novel materials have become critical processes to meet these requirements in developing TMO‐based composite electrodes,^1^, ^6^, ^17^, ^23^, ^25^ wherein various conductive materials, including nanostructured carbons (such as porous carbon,^14^, ^26^ carbon nanotubes,^27^, ^28^, ^29^, ^30^ and graphene 31, 32) and conducting polymers, are extensively employed to serve as electron pathways. Although the large specific surface area in these low‐dimensional composite nanostructures allows ion transports,^1^, ^17^, ^23^, ^26^, ^27^, ^28^, ^29^, ^30^, ^31^, ^32^ their assembled bulk electrodes usually exhibit high electrical resistance as a result of the short electron transport distance within these low‐dimensional conductive materials, the undesirably high contact resistances produced by the coating of electrically insulating active TMOs and polymer binders, as well as the weak and noncoherent TMO/conductor interfaces.^24^, ^33^, ^34^, ^35^ This inevitably leads to unsatisfactory improvements in rate capability, volumetric energy and power densities of pseudocapacitors.^1^, ^17^, ^23^ Therefore, it is imperative to explore novel and low‐cost conductive electrode materials that can make their composite electrodes deliver more volumetric energy at high rates with long‐term cycling stability by tackling all the three of abovementioned problems.

Here we look beyond conventional metals and carbon materials and search for promising electrode materials in the reservoir of strongly correlated electron systems of TMOs in view of their interesting physical properties,^36^, ^37^ including insulator‐to‐superconductor and insulator‐to‐metal transitions (IMTs) accompanied by huge resistivity changes at their transition temperatures.^38^ Unlike transitions to superconductivity only taking place below the critical transition temperature, IMTs happen as temperature increases and thus enable these strongly correlated materials to behave metallic state in their high‐temperature phases,^36^, ^37^, ^38^, ^39^ which is expected to have practical applications. In this work, we report a classic strongly correlated TMO, vanadium sesquioxide (V_2_O_3_), with a 3D bicontinuous nanoporous architecture (NP V_2_O_3_) as a conductive network penetrating in all‐ceramic hybrid electrodes of V_2_O_3_/MnO_2_ (NP V_2_O_3_/MnO_2_) for high‐performance pseudocapacitors. Therein, the 3D NP V_2_O_3_ core skeleton with a corundum‐type crystalline structure becomes highly conductive at ambient temperature as a consequence of metallization via IMT.^38^, ^39^ The interpenetrating nanopores provide not only fast ion transport channels but abundant V_2_O_3_/MnO_2_ epitaxial interfaces that dramatically enhance both the interfacial electron transfer via V—O—Mn chemical bonding and the electrical conductivity of the insulting MnO_2_ layer by the formation of semiconductive region. These merits grant the constituent MnO_2_ sandwiched between highly efficient electron and ion pathways taking full advantage of its high theoretical pseudocapacitance even at rapid charge/discharge rates in pseudocapacitors. As a result, the symmetric pseudocapacitors based on the additive‐free V_2_O_3_/MnO_2_ electrodes deliver volumetric power densities (up to ≈422 W cm^−3^) comparable to those of 3 V–30 μF Al electrolytic capacitor, without compromising cyclability and energy reduction.

## 2 Results and Discussion

**Figure 1:**
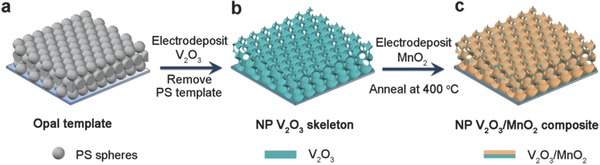
Schematic illustrating fabrication of heterostructured electrodes. a) NH_4_^+^‐terminated PS spheres are self‐assembled to an opal film on stainless steel substrate via evaporative deposition at 80 °C. b) 3D bicontinuous nanoporous V_2_O_3_ film is formed directly on the SS substrate by electrodeposition of vanadium oxide and selective removal of PS opal template followed by calcination at 450 °C in H_2_/Ar atmosphere. c) MnO_2_ nanocrystals are decorated onto nanoporous channels of V_2_O_3_ skeleton by pulse electrodeposition in a mixed aqueous solution containing 50 × 10^−3^ m MnSO_4_.

**Figure 2:**
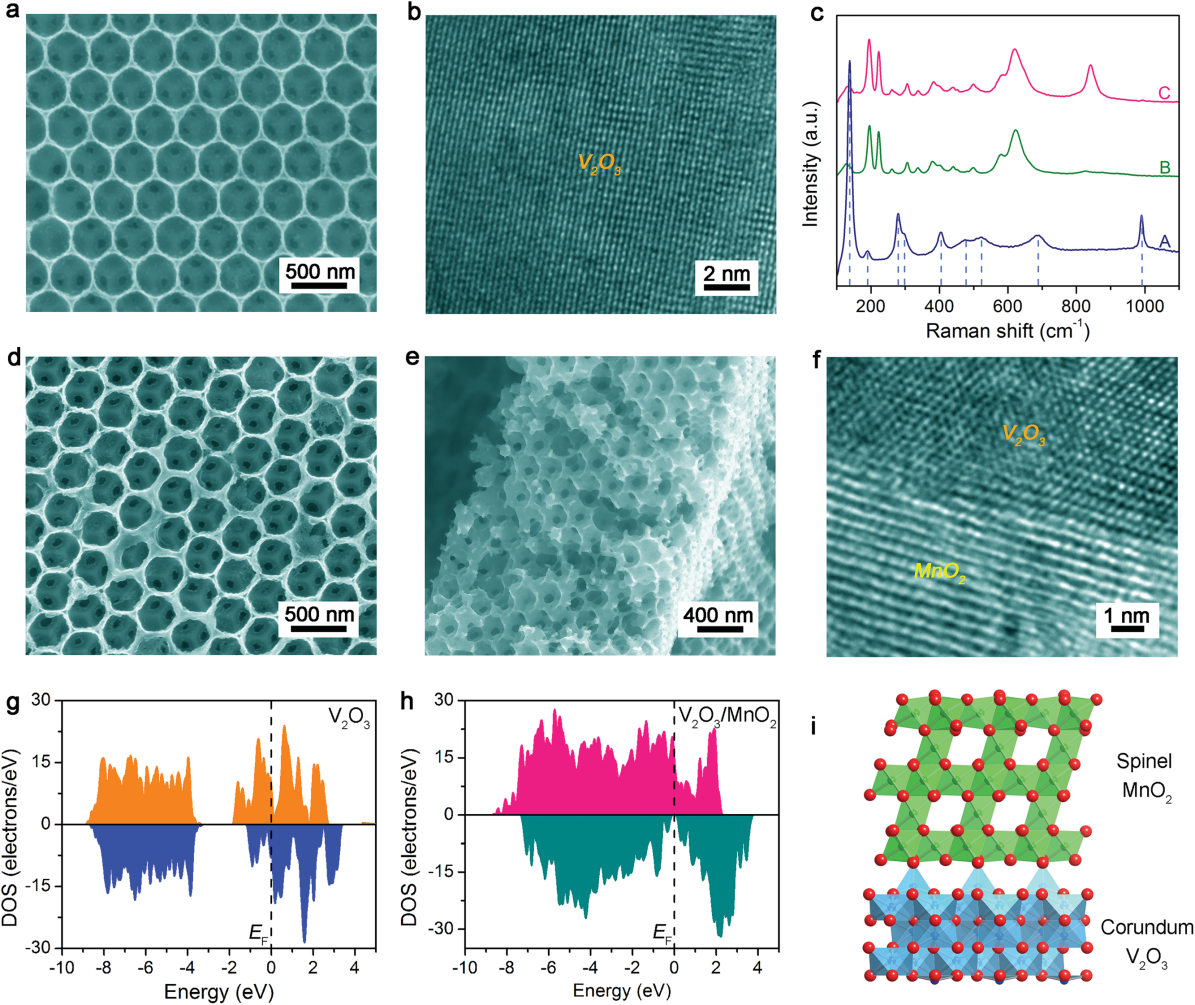
Microstructure characterization and theoretical calculation. a) SEM image of 3D nanoporous V_2_O_3_ scaffold supported on SS substrate after removal of PS opal template. b) HRTEM image of corundum‐type crystalline V_2_O_3_ skeleton. c) Raman spectra of A) V_2_O_3_, and V_2_O_3_/MnO_2_ B) before and C) after heat treatment at 400 °C. d) Top‐view and e) cross‐section SEM images of nanoporous V_2_O_3_/MnO_2_ hybrid electrode with an MnO_2_ pulse‐electrodeposition time of 110 s. f) HRTEM image of V_2_O_3_/MnO_2_ interfacial structure. Total spin‐polarized densities of states (DOS) of g) the corundum V_2_O_3_ bulk and h) the V_2_O_3_/MnO_2_ interface. Positive DOS is for spin up and negative is for spin down. The dashed line indicates the position of Fermi level *E*_F_. i) Periodic superlattice of interface between corundum V_2_O_3_(0001) layers and spinel MnO_2_(111) layers.

Our strategy to fabricate porous electrodes of heterostructured electron‐correlated oxides makes use of periodic opal and inverse opal templates,^24^, ^40^ on which vanadium and manganese oxides are consecutively electrodeposited to produce 3D bicontinuous NP V_2_O_3_/MnO_2_ electrodes (see schematical diagram illustrated in **Figure** **1** and Experimental Section). NH_4_^+^‐terminated polystyrene (PS) latex nanospheres with diameter of ≈450 nm are firstly synthesized by soapless dispersion polymerization 41 and then self‐assembled on stainless steel (SS) sheets via evaporative deposition for the production of the periodic opal templates (Figure S1, Supporting Information).^42^ After the electrodeposition of vanadium oxide through these opal templates in a mixed solution containing VOSO_4_, the inverse opal films of V_2_O_3_ are prepared by calcination at 450 °C in H_2_/Ar air, during which the PS nanospheres are selectively removed and the vanadium oxide is thermally reduced. These bare NP V_2_O_3_ films composed of polycrystalline domains are crack‐free, and more importantly, the nanopores at the surface are open (Figure S2, Supporting Information). **Figure** **2**a shows representative top‐view scanning electron microscope (SEM) image of the V_2_O_3_ skeletons in a domain, demonstrating a uniform and 3D bicontinuous nanoporous structure that consists of periodic walls and open nanopores with characteristic length scales of ≈28 and ≈360 nm, respectively (Figure S3, Supporting Information). High‐resolution transmission electron microscope (HRTEM) image reveals the crystalline nature of the V_2_O_3_ network (Figure 2b), in which an interplanar spacing of ≈0.278 nm corresponds to the distance of V–V pairs in (0001) plane of a corundum‐type crystalline structure generated by the structure change from monoclinic insulating state at IMT.^38^, ^39^, ^43^ The corundum structure of V_2_O_3_ at ambient temperature is further substantiated by Raman spectrum (blue curve in Figure 2c) and X‐ray diffraction pattern (Figure S4a, Supporting Information). The well‐resolved diffraction peaks correspond to the (012), (104), (110), (113), (024), (116), (214), and (300) planes of corundum‐type of V_2_O_3_ (JCPDS 34–0187), apart from two obvious diffraction peaks at 2*θ* = 43.3° and 50.6° attributed to SS substrate (Figure S4a, Supporting Information).

Pseudocapacitive MnO_2_ nanocrystals are incorporated into the conductive 3D NP V_2_O_3_ skeletons by pulsed electrodeposition in a mixture of MnSO_4_ and Na_2_SO_4_, and the loading mass of MnO_2_ can be controlled by adjusting the anodic deposition time (Table S1, Supporting Information). Considering that the electrical property and electrochemical performance of TMOs can be improved by the introduction of heteroatoms or intrinsic defects,^6^, ^44^ the as‐prepared NP V_2_O_3_/MnO_2_ films are subsequently exposed in H_2_/Ar at 400 °C. This process maintains the chemical states of V_2_O_3_ scaffold and hydrogenates the MnO_2_ layer,^44^ which are verified by X‐ray photoelectron spectroscopy (XPS) survey (Figure S5a–c, Supporting Information). As a result of the introduction of oxygen vacancies by hydrogenation, the Mn 3s core level XPS spectrum displays a large energy separation of 5.5 eV. Representative top‐view SEM image of NP V_2_O_3_/MnO_2_ architecture illustrates that the electrodeposition process affords MnO_2_ nanocrystals uniformly grown along the walls of ≈2 μm thick V_2_O_3_ scaffold (Figure 2d,e) with evident size change of walls and nanopores (Figure S3b, Supporting Information). In the XRD patterns of NP V_2_O_3_/MnO_2_, the peaks at 2*θ* = 18.5°, 30.9°, and 36.1° correspond to the (111), (220), and (311) plane of spinel‐type MnO_2_, in addition to the characteristic peaks of NP V_2_O_3_ scaffold with depressed intensity, which is due to the coating of MnO_2_ layer with poor crystallinity (Figure S4b, Supporting Information). The interfacial structure of V_2_O_3_/MnO_2_, as shown in the HRTEM image (Figure 2f), indicates the epitaxial growth of spinel MnO_2_ nanocrystals on V_2_O_3_ network surfaces with the assistance of stabilizing Na^+^ cation and the further formation of chemical V—O—Mn bonding at the interface during the heat treatment process.^45^, ^46^ As a consequence, the Raman spectrum of the heat‐treated NP V_2_O_3_/MnO_2_ electrode exhibits a characteristic Raman peak at 842 cm^−1^ with dramatically enhanced intensity (pink curve in Figure 2c),^46^ in addition to peaks from both corundum V_2_O_3_ and spinel MnO_2_ in the pristine one (green curve in Figure 2c). Furthermore, the Raman spectra of the annealed and pristine electrodes retain the same in the feature range from 100 cm^−1^ to 750 cm^−1^, showing no crystalline structure changes during the annealing process. The compelling evidence of V—O—Mn interface structure is further elucidated by density functional theory (DFT) calculations on a periodic supercell of corundum V_2_O_3_(0001)/spinel MnO_2_(111) multilayer.^47^ The Hirshfeld charge state of the interfacial V atoms increases to ≈0.500 e from ≈0.330 e of the bare V_2_O_3_ (Figure S6, Supporting Information), implying that the chemical state of partial V atoms at the V_2_O_3_/MnO_2_ interface changes from V^3+^ to V^4+^, as shown in the V 2p XPS spectrum with their characteristic peaks of V 2p_1/2_ and 2p_3/2_ at the binding energies of 522.8, 523.9, 515.5, and 516.6 eV, respectively (Figure S5d, Supporting Information).^48^, ^49^ In comparison with the density of electronic states (DOS) for the corundum V_2_O_3_ bulk (Figure 2g), a continuous valence band is produced in the heterostructured V_2_O_3_(0001)/MnO_2_(111) interface due to the formation of chemical V—O—Mn bonding between the surface‐terminated V atoms of the conductive V_2_O_3_(0001) and the three‐coordinated O surface atoms in the spinel MnO_2_(111) (Figure 2 h,i, and Figure S7a in the Supporting Information). Moreover, the Fermi energy lies in the nonzero DOS, demonstrating that the V_2_O_3_(0001)/MnO_2_(111) interface is metallic. To evaluate the evolution of electron transport capability in MnO_2_ multilayer as a function of layer number away from V_2_O_3_, the states of separate Mn‐O layers are calculated by integrating their local DOS (LDOS) near the Fermi energy over an energy window of 400 meV (Figure S7b, Supporting Information). The remarkable charge transfer at the V—O—Mn interface triggers metallic V_2_O_3_ induced energy levels to cross the Fermi energy for each Mn—O layer (Figure S7b, Supporting Information), and the number of states gradually decreases and then remains a constant with the further increase of layer number, implying more or less states near the Fermi energy that are available for transport. The whole MnO_2_ multilayer in the vicinity of V_2_O_3_ therefore exhibits a strikingly enhanced conductivity compared with the bare one (Figure S8, Supporting Information), confirmed by the current–voltage (*I*–*V*) measurements of the NP V_2_O_3_/MnO_2_ electrodes. As shown in Figure S9 (Supporting Information), the NP V_2_O_3_/MnO_2_ electrodes display a linear *I*–*V* curve with a resistance of 12.8 Ω (resistivity of ≈2.6 × 10^−3^ Ω cm) above the external voltage of 0.4 V, in accordance with that of conductive V_2_O_3_ scaffold (≈395 S cm^−1^). The good conductivity ensures an efficient electron transport of NP V_2_O_3_/MnO_2_ for high‐rate charge and discharge.

**Figure 3:**
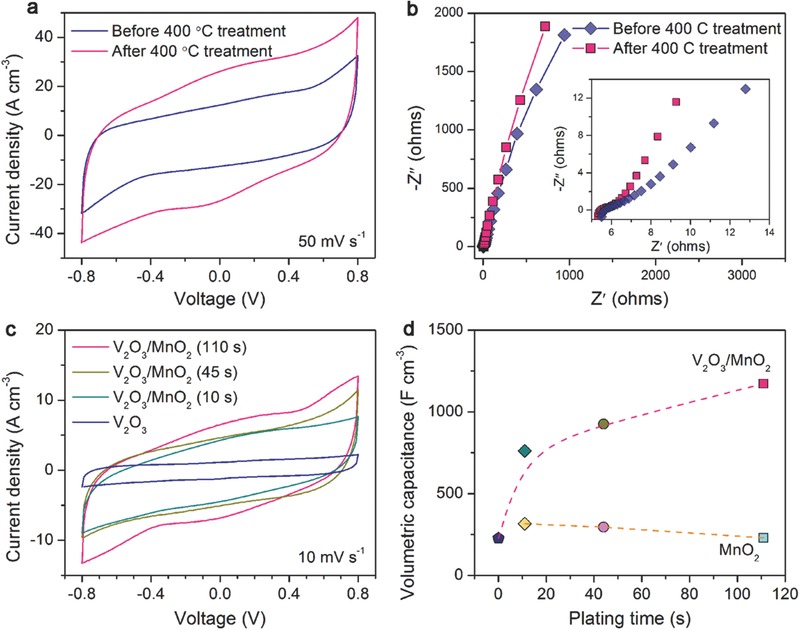
Electrochemical characterization. a) Comparative cyclic voltammetry (CV) curves of V_2_O_3_/MnO_2_ electrodes (plating time, 110 s) before and after H_2_ reduction at 400 °C. Scan rate, 50 mV s^−1^. b) Complex plane plot of the impedance of V_2_O_3_/MnO_2_ electrode before and after additional heat treatment. Inset: a magnification for the high‐frequency region. c) CV curves for bare nanoporous V_2_O_3_ electrodes and nanoporous V_2_O_3_/MnO_2_ hybrid electrodes with three different plating times. Scan rate, 10 mV s^−1^. d) Volumetric capacitances of NP V_2_O_3_/MnO_2_ electrodes and NP MnO_2_ supported by SS substrates as a function of plating time at 10 mV s^−1^. All electrochemical data are collected on symmetric two‐electrode supercapacitors in a 1 m Na_2_SO_4_ aqueous electrolyte at room temperature.

To assess the pseudocapacitive properties, electrochemical measurements for the NP V_2_O_3_/MnO_2_ electrodes are performed in symmetric two‐electrode configuration in 1 m Na_2_SO_4_ aqueous electrolyte. **Figure** **3**a shows typical cyclic voltammetric (CV) curves collected at a scan rate of 50 mV s^−1^ for the NP V_2_O_3_/MnO_2_ electrodes (plating time, 110 s) before and after exposure to the H_2_/Ar (5%) atmosphere at 400 °C. For comparison, the CV curve of the NP V_2_O_3_/MnO_2_ electrodes heat‐treated in the pure Ar atmosphere at 400 °C is plotted in Figure S10a (Supporting Information). All CV curves exhibit symmetrical rectangular shapes in a voltage window from −0.8 to 0.8 V.^50^, ^51^ Extraordinarily, the NP V_2_O_3_/MnO_2_ electrodes heat‐treated in the H_2_/Ar (5%) atmosphere displays the highest current density than those of the heat‐treated ones with pure Ar protection and the pristine ones, which originates from the hydrogenation of MnO_2_ layer and the formation of chemical V—O—Mn bonding at the epitaxial V_2_O_3_/MnO_2_ interface during annealing. This suggests their significant roles in improving the pseudocapacitive behavior of the NP V_2_O_3_/MnO_2_ films: It not only intrinsically improves the electronic conductivity of pseudocapacitive MnO_2_ layer (Figure S7 and S8, Supporting Information) but also facilitates the charge transfer from the MnO_2_ layer to the conductive V_2_O_3_ skeleton (Figure 2h). The high electron transport pathway by interconnective V_2_O_3_ network and the fast ion transport channels by the interpenetrating nanopores facilitate the charge/discharge processes in of the NP V_2_O_3_/MnO_2_ electrodes at a pseudo‐constant rate over the voltammetric cycles. Therein, the entire constituent MnO_2_ layer sandwiched between highly conductive ion and electron transport pathways participates in the processes of both the surface electrosorption of Na^+^ cations and the fast, reversible surface redox reaction, i.e., MnO_2_ + *a*H^+^ + *b*Na^+^ + (*a*+*b*)e^−^ ↔ MnOOH*_a_*Na*_b_*.^1^, ^2^, ^3^, ^15^, ^20^, ^52^ The enhanced pseudocapacitive behavior is further confirmed by comparison of electrochemical impedance spectroscopy (EIS) spectra for NP V_2_O_3_/MnO_2_ electrodes before and after heat treatments (Figure 3b, and Figure S10b in the Supporting Information). At high frequencies, almost the same intercepts of the Nyquist curves with the real axis imply the constant solution resistance in both electrodes.^3^, ^25^, ^53^ In the middle‐frequency region, the negative shift of the semicircle for the heat‐treated NP V_2_O_3_/MnO_2_ electrodes denotes that their lower charge‐transfer resistance than the pristine ones (inset of Figure 3b). This also gives rise to an enhanced capacitive behavior indicated by a pronounced increase of the imaginary part of EIS at low frequency.^3^, ^25^, ^53^

Figure 3c shows the CV curves of the heat‐treated NP V_2_O_3_/MnO_2_ electrodes at a scan rate of 10 mV s^−1^ as a function of electroplating time of MnO_2_. With the loading and hydrogenating of pseudocapacitive MnO_2_, the volumetric current density of NP V_2_O_3_/MnO_2_ electrodes becomes much higher than that of the bare NP V_2_O_3_ skeletons, demonstrating that the capacitance can be significantly improved by incorporating pseudocapacitive material with a high theoretical capacity into the 3D nanoporous structure. The increment of volumetric capacitance depends on the loading amount of the pseudocapacitive MnO_2_ and increases with the electroplating time (Figure 3d). When the mass ratio of MnO_2_ increases to ≈47 wt%, a volumetric capacitance as high as ≈1172 F cm^−3^ is achieved at a scan rate of 10 mV s^−1^ for the NP V_2_O_3_/MnO_2_ with thickness of 2 μm, comparable to 1160 F cm^−3^ for 100 nm‐thick NP Au/MnO_2_ ultrathin films at a scan rate of 50 mV s^−1^ 54 and much higher than some of the highest reported previously: 900 F cm^−3^ for Ti_3_C_2_T_x_ clay electrodes at a scan rate of 2 mV s^−1^,^7^ 78.6 F cm^−3^ for MnO_2_/Au multilayers at a scan rate of 10 mV s^−1^,^25^ 246 F cm^−3^ for layer‐by‐layered MWNT/MnO_2_ electrodes at a scan rate of 10 mV s^−1^,^28^ and 71.6 F cm^−3^ for reduced graphene films at 10 mV s^−1^.^10^ For comparison, the volumetric capacitances of MnO_2_ nanocrystals electrodeposited on SS sheets with different time and heat‐treated at the same H_2_/Ar atmosphere are also included in Figure 3d. Despite the nanoporous structure, the capacitive performance of the MnO_2_ films follows a general observation that the thicker the electro‐active films, the lower the volumetric capacitance,^15^, ^52^ greatly inferior to that of the NP V_2_O_3_/MnO_2_ electrodes. This distinct contrast further verifies the advantages of the 3D bicontinuous NP V_2_O_3_ skeleton as a conductive scaffold in enhancing the pseudocapacitive performance of the constituent MnO_2_, i.e., the nanosized conductive V_2_O_3_ affords abundant epitaxial interfaces to facilitate fast electron transfer between MnO_2_ and V_2_O_3_; the nanoporous channels accelerate ion transport and enable sufficient contact between the supported MnO_2_ and electrolyte for approaching its high theoretical pseudocapacitance.

**Figure 4:**
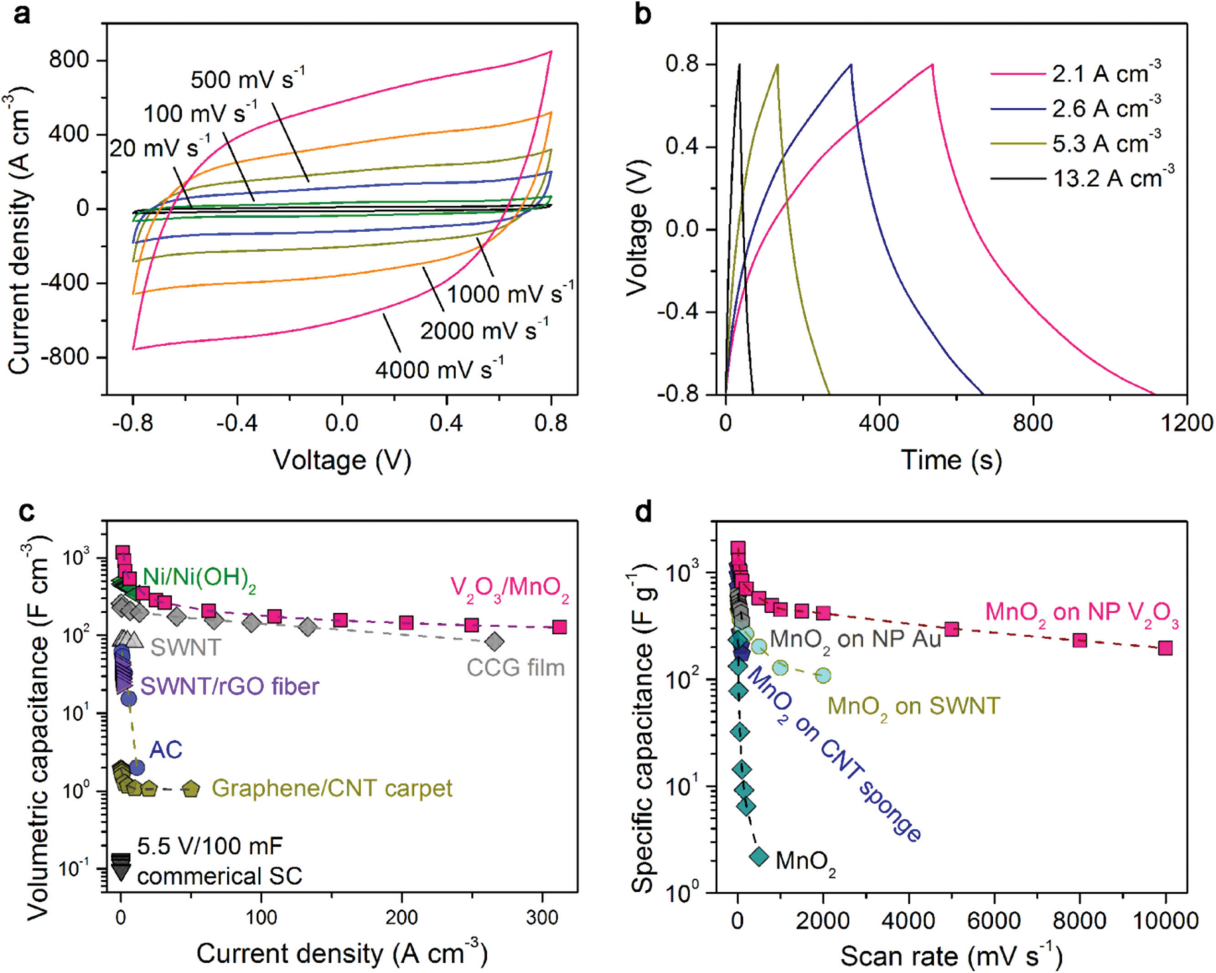
Performance of V_2_O_3_/MnO_2_‐based electrochemical pseudocapacitors. a) CV curves for pseudocapacitors assembled with NP V_2_O_3_/MnO_2_ electrodes (plating time, 110 s) in 1 m Na_2_SO_4_ electrolyte at various scan rates. b) Galvanostatic charge/discharge profiles at various current densities for NP V_2_O_3_/MnO_2_ based pseudocapacitors. c) Volumetric capacitance of NP V_2_O_3_/MnO_2_ electrodes as a function of different current densities, comparing with these of SWNT/rGO fibers,^11^ graphene/CNT carpets,^12^ liquid‐mediated CCG film,^13^ SWNTs,^14^ AC,^14^ NP Ni/Ni(OH)_2_,^39^ and commercial supercapacitor (5.5 V/100 mF SC).^11^ d) Specific capacitance of the constituent MnO_2_ in the NP V_2_O_3_/MnO_2_ hybrid electrode as a function of scan rate, in comparison with that of NP MnO_2_ supported by CNT sponges,^29^ SWNT films,^30^ NP Au,^57^ and stainless steel substrate.

For the typical pseudocapacitor that is assembled by NP V_2_O_3_/MnO_2_ electrodes with the highest loading amount of MnO_2_ (47 wt%) in a symmetric two‐electrode device, the CV curves collected at scan rates from 5 to 10 000 mV s^−1^ are shown in **Figure** **4**a and Figure S11a (Supporting Information). As a result of the unique nanoarchitecture with enhanced ion and electron transport kinetics in NP V_2_O_3_/MnO_2_ electrodes, the CV curves of pseudocapacitor have an almost rectangular shape at scan rates below 4000 mV s^−1^ and remain quasi‐rectangular at scan rates up to 10 000 mV s^−1^. A linear dependence of the discharge current at 0.2 V on the scan rate up to 4000 mV s^−1^ indicates a surface‐redox limited process (Figure S11b, Supporting Information), which enables the excellent charge storage characteristics of the NP V_2_O_3_/MnO_2_ electrodes without any depression of voltammetric response compared with that of the bare NP V_2_O_3_ framework (Figure S12, Supporting Information). Their galvanostatic charge and discharge curves at various current densities show a curvature (Figure 4b), implying the mainly pseudocapacitive contribution to the total capacitance in the NP V_2_O_3_/MnO_2_ electrodes, in addition to the double‐layer capacitance. The small voltage drop at the beginning of the each discharge results from the low equivalent series resistance of ≈12 Ω in the aqueous electrolyte (Figure S13, Supporting Information), in accordance with *I*–*V* and EIS measurements. Figure 4c presents the volumetric capacitance of the NP V_2_O_3_/MnO_2_ electrodes in a wide range of charge/discharge current densities from 1.56 to 312 A cm^−3^. The NP V_2_O_3_/MnO_2_ electrodes exhibit the highest volumetric capacitance of ≈1162 F cm^−3^ at 1.56 A cm^−3^. Despite slight decrease of the volumetric capacitance with the increase of the current density up to up to 312 A cm^−3^, the NP V_2_O_3_/MnO_2_ based pseudocapacitor can sustain high volumetric capacitances at ultrafast charge/discharge rates, which are ≈2–5 times higher than the best values reported for EDLCs based on the chemically converted graphene (CCG) films with a high packing density of 1.33 g cm^−3^ (Figure 4c).^13^ This excellent rate capability is also much better than those of other carbon materials, such as single‐walled carbon nanotubes/reduced graphene oxide (SWNT/rGO) fibers,^11^ graphene/carbon nanotube (graphene/CNT) carpet,^12^ SWNTs^14^ or activated carbon (AC),^14^ and the pseudocapacitive Ni(OH)_2_ supported by NP Ni skeleton [NP Ni/Ni(OH)_2_].^55^, ^56^

To evaluate the contribution of the electroactive MnO_2_ to the electrochemical performance of the NP V_2_O_3_/MnO_2_ electrodes, the specific capacitance of the constituent MnO_2_ (*C*_s,MnO2_) is calculated after subtracting the charge of the bare NP V_2_O_3_ framework according to the equation *C*_s,MnO2_ = (*Q*_V2O3/MnO2_ − *Q*_V2O3_)/(Δ*Em*_MnO2_). Here *Q*_V2O3/MnO2_ and *Q*_V2O3_ are the voltammetric charges derived from CV curves of the NP V_2_O_3_/MnO_2_ and NP V_2_O_3_ electrodes at various scan rates (Figure 4a, and Figures S11 and 12 in the Supporting Information), respectively, *m*_MnO2_ is the mass of MnO_2_ and Δ*E* is the potential window. As shown in Figure 4d, the specific capacitance of the constituent MnO_2_ increases with the decreasing scan rate, and reaches ≈1301 F g^−1^ at 20 mV s^−1^, very close to its theoretical value of specific capacitance (≈1375 F g^−1^) due to both pseudocapacitive and double‐layer capacitive contributions in the highly utilized electrode materials. More impressively, when the scan rate is increased to 10 000 mV s^−1^, the specific capacitance can maintain ≈195 F g^−1^. This is far superior to the rate performances of the MnO_2_ nanocrystals supported by CNT sponges,^29^ SWNT films,^30^ or NP Au skeleton,^57^ as well as that of the nanoporous MnO_2_ directly grown on SS sheets and heat‐treated in H_2_/Ar (5%) atmosphere at 400 °C, where their specific capacitance dramatically decreases with the increasing scan rates (Figure 4d).

**Figure 5:**
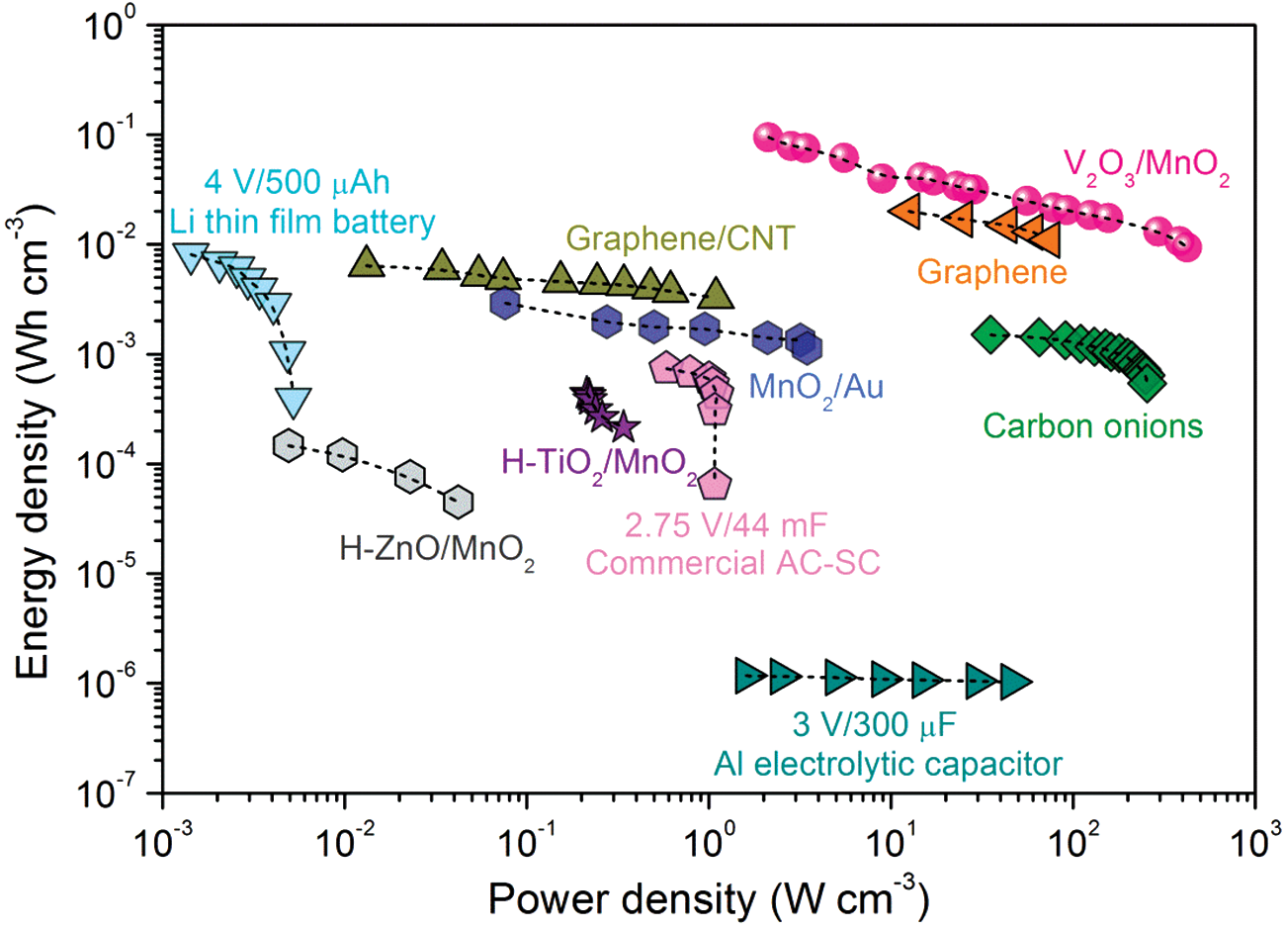
Ragone plot of energy storage devices. The comparison of energy and power densities of NP V_2_O_3_/MnO_2_‐based pseudocapacitors with EDLCs based on carbon onions,^8^ SWNT/rGO fibers,^11^ liquid‐mediated dense graphene,^13^ and pseudocapacitors assembled with H‐TiO_2_/MnO_2_,^6^ H‐ZnO/MnO_2_,^20^ MnO_2_/Au multilayers,^25^ as well as commercial devices such as lithium thin‐film batteries,^8^, ^11^ 2.75/44 mF AC supercapacitor (AC‐SC),^9^ and conventional 3 V/300 μF Al electrolytic capacitor.^11^

The volumetric power and energy densities of NP V_2_O_3_/MnO_2_‐based pseudocapacitor are plotted in the Ragone plot (**Figure** **5**), in which the values of other energy storage devices, such as carbon‐based EDLCs^8^, ^11^, ^13^ and TMO‐based pseudocapacitors,^6^, ^20^, ^25^ as well as commercial AC supercapacitor^9^ and electrolytic capacitor,^11^ are included for comparison. At low powers (2.1 W cm^−3^), the pseudocapacitor assembled with the NP V_2_O_3_/MnO_2_ electrodes has a volumetric energy density of ≈94 mWh cm^−3^, which is two orders of magnitude higher than that of commercially available 2.75 V/44 mF AC supercapacitor.^9^ Furthermore, this value is also much higher than the highest values recently reported in EDLCs based on carbon onions,^8^ graphene/CNT hybrid^11^ or graphene,^13^ and pseudocapacitors generated with the hydrogenated TiO_2_/MnO_2_ (H‐TiO_2_/MnO_2_/C)^6^ or ZnO/MnO_2_ (H‐ZnO/MnO_2_/C)^20^ supported by carbon cloths, or MnO_2_/Au multilayers.^25^ The maximum volumetric power density can reach ≈422 W cm^−3^ with the energy density of ≈9.3 mWh cm^−3^, ≈700‐fold higher than that of a commercial AC supercapacitor (2.75 V/44 mF)^9^ and more than five orders of magnitude higher than that of lithium thin‐film battery (4 V/500 μAh) with the energy density of ≈8 mWh cm^−3^.^8^ To our knowledge, this volumetric power density is the highest value among all TMO‐based pseudocapacitors reported to date. Cycling life tests over 15 000 cycles for the NP V_2_O_3_/MnO_2_‐based pseudocapacitor are performed at a scan rate of 500 mV s^−1^ and the results are shown in Figure S14 (Supporting Information). In spite of a slight reduction, the capacitance retains ≈86% of the initial value after the long‐term electrochemical cycling, demonstrating the excellent stability of the hybrid electrodes.

The superior electrochemical performance of NP V_2_O_3_/MnO_2_‐based pseudocapacitors originates from the combination of faradaic and non‐faradaic processes that take place in the highly conductive V_2_O_3_/MnO_2_ heterostructure with 3D bicontinuous nanoporous core/shell architecture.^22^ Because of metallization via a first‐order IMT above the transition temperature, V_2_O_3_ behaves as a half‐filled metal with a 3*d* ^2^ state at ambient temperature.^38^, ^39^, ^43^ Therefore, the 3D bicontinuous V_2_O_3_ skeleton works as a conductive scaffold for the incorporation of pseudocapacitive MnO_2_ nanocrystals. The nanosized V_2_O_3_ skeleton not only provides interconnected nanopore channels for the accelerated ion transports but also offers abundant V_2_O_3_/MnO_2_ epitaxial interfaces with the chemically stable V—O—Mn bonding, which dramatically improves the electronic conductivity of MnO_2_ layer and thus expedites the charge transfer at interface region. Although MnO_2_ has intrinsically low conductivity that limits its charge/discharge rate.^1^, ^6^, ^15^, ^17^, ^23^ These microstructural virtues ensure the full use of the high theoretical pseudocapacitance of MnO_2_ layer with the exceptionally pseudocapacitive performance, ultrahigh rate capability, and excellent cycling stability. Moreover, the V_2_O_3_ core of the porous hybrid composite further contributes to the high‐density energy storage by a fast and reversible surface redox reaction with a specific capacitance much higher than those EDLCs of conventional conductive reinforcements such as nanostructure metals or carbon materials (Figure S12, Supporting Information).^1^, ^54^ Consequently, these dual pseudocapacitive mechanisms give rise to ultrahigh volumetric energy of the all‐ceramic hybrid electrodes delivered at exceptionally high power densities with a long‐term electrochemical stability.

## 3 Conclusion

In summary, we have developed novel heterostructured V_2_O_3_/MnO_2_ electrodes with a 3D bicontinuous and nanoporous architecture as promising electrode materials for high‐performance electrochemical pseudocapacitors. Owing to its insulator‐to‐metal transition, the 3D bicontinuous nanoporous skeleton of strongly correlated V_2_O_3_ system becomes conductive at ambient temperature, having the constituent pseudocapacitive MnO_2_ layer sandwiched between high pathways of electrons and ions for the full utilization of high theoretical pseudocapacitance. This allows the pseudocapacitor based on the V_2_O_3_/MnO_2_ hybrid electrodes to deliver exceptionally high volumetric power density (≈422 W cm^−3^), ≈380‐fold higher than that of a commercial AC supercapacitor (2.75 V/44 mF) and more than five orders of magnitude higher than that of lithium thin‐film battery (4 V/500 μAh), along with excellent cycling stability over 15 000 cycles. The volumetric energy density up to ≈94 mWh cm^−3^ is one order of magnitude higher than that of lithium thin‐film battery (4 V/500 μAh). The impressive pseudocapacitive energy storage/delivery performance results from the unique nanoarchitecture of heterostructured NP V_2_O_3_/MnO_2_ electrodes with plentifully epitaxial interfaces of chemical V–O–Mn bonding, and makes them promising candidates as electrode materials in next‐generation electrochemical capacitors.

## 4 Experimental Section

*Fabrication of NP V_2_O_3_ and V_2_O_3_/MnO_2_ Electrodes*: The ordered NP V_2_O_3_ and V_2_O_3_/MnO_2_ heterostructured electrodes with size of 0.4 cm × 0.4 cm × 2 μm were constructed directly on SS current collectors by using a polystyrene (PS) opal template. The NH_4_^+^‐terminated PS latex particles with a mean diameter of ≈450 nm were synthesized by a soapless dispersion polymerization approach in a mixed ionic/nonionic initiation system,^41^ and then formed to an opal film on the SS substrates via evaporative deposition at 80 °C.^42^ Vanadium oxide was electrodeposited into the PS opal templates using a classic three‐electrode setup at 1.5 V for 100 s in a mixture of 1 m VOSO_4_, 1 × 10^−3^ m H_2_SO_4_, 40 mL pure water, and 50 mL ethanol. Here a platinum foil and an Ag/AgCl electrode were employed as the counter electrode and the reference electrode, respectively. The 3D bicontinuous nanoporous V_2_O_3_ scaffold electrodes were obtained by calcining the films in H_2_/Ar air at 450 °C for 10 h to remove the PS template.MnO_2_ was further incorporated on the NP V_2_O_3_ skeleton by using pulsed electrodeposition in an aqueous solution containing 50 × 10^−3^ m MnSO_4_ and Na_2_SO_4_ for different time (Table S1, Supporting Information). The samples were rinsed with deionized water to remove the salt residue and annealed at 400 °C for 2 h in a tube furnace under a flowing atmosphere (5% H_2_ in Ar). The MnO_2_/SS foils were prepared by electrodepositing bare NP MnO_2_ on SS substrate in the same electrolyte and annealing conditions.

*Characterizations*: The microstructure and chemical composition of the specimens were investigated using a field‐emission transmission electron microscope (JEOL JEM‐2100F, 200 keV), and a field‐emission scanning electron microscope (JEOL JSM‐6700F, 15 keV) equipped with an X‐ray energy‐dispersive spectroscopy. Raman spectra were collected using a micro‐Raman spectrometer (Renishaw) with a laser of 532 nm wavelength at 0.2 mW. X‐ray diffraction measurement was carried out on a D/max2500pc diffractometer using Cu Kα radiation.

*Electrochemical Measurement*: Symmetric pseudocapacitors were assembled with two pieces of SS‐supported NP V_2_O_3_, or V_2_O_3_/MnO_2_ electrodes, and cotton paper as a separator. To characterize the electrochemical performance of the supercapacitor devices, all electrochemical energy storage behaviors were evaluated in a two‐electrode setup (Iviumstat electrochemical analyzer, Ivium technology) in 1 m Na_2_SO_4_ aqueous electrolyte. Cyclic voltammetry and galvanostatic charge/discharge were performed in a potential window from −0.8 to 0.8 V at various scan rates and current densities, respectively. Electrochemical cycling stabilities were tested using cyclic voltammetry experiments with a scan rate of 500 mV s^−1^ for over 10 000 cycles. The volumetric capacitance (*C*_V_) was calculated by integrating a segment of the cyclic voltammograms according to Equation (1), 8, 10, 11

(1) $$C_{V}\;=\;Q/(\Delta EV),$$

where *Q* is the voltammetric charge in the potential region Δ*E* = 1.6, *V* is the geometric volume of NP V_2_O_3_, or V_2_O_3_/MnO_2_ electrode with size of 0.4 cm × 0.4 cm × 2 μm. *v* is the scan rate and *I*(*E*) is the current as a function of potential *E*. The volumetric power (*P*_V_) and energy densities (*W*_V_) were calculated in terms of Equations (2) and (3), 8, 10, 11

(2) $$P_{V}\;=\;\int_{0}^{0.8}I(E)dE/V,$$

(3) $$W_{V}\;=\;\Delta E\int_{0}^{0.8}I(E)dE/\left(3600\nu V\right).$$

While the *C*_V_ of NP V_2_O_3_/MnO_2_ electrodes evaluated by using the charge/discharge curves was calculated according to *C*_V_ = *i*/[−(Δ*E*/Δ*t*)*V*] with *i* being the applied current, Δ*E*/Δ*t* the slope of the discharge curves.

*Calculation Method*: First‐principles electronic structure calculations were performed within density functional theory on the periodic supercells of corundum V_2_O_3_(0001)/spinel MnO_2_(111), bulk corundum V_2_O_3_ and spinel MnO_2_ systems,^47^ respectively, using the CASTEP code with the ultrasoft pseudopotentials. The exchange‐correlation effects were described by the local density approximation (LDA) with spin polarization. The calculations were carried out using a plane‐wave basis set with a cutoff energy of 400 eV and the 5 × 5 × 1 mesh of *k* points was set in the Brillouin zone. Atomic relaxations were performed until energy, maximum force, and maximum displacement have become less than 10^−5^ eV atom^−1^, 0.03 eV Å^−1^, and 0.001 Å, respectively. A 16 Å thick vacuum was added along the direction perpendicular to the interface of V_2_O_3_/MnO_2_. The electronic structures of spinel MnO_2_ were calculated within the LDA+U approach that involves electron–electron correlations. The value of Hubbard U (3.3 eV) was employed to demonstrate a reasonable electronic structure of bulk spinel MnO_2_ for the partially filled Mn 3*d* states.

## Supporting information

As a service to our authors and readers, this journal provides supporting information supplied by the authors. Such materials are peer reviewed and may be re‐organized for online delivery, but are not copy‐edited or typeset. Technical support issues arising from supporting information (other than missing files) should be addressed to the authors.

Supplementary
