# Supplementary material for: Ultrahigh‐Power Pseudocapacitors Based on Ordered Porous Heterostructures of Electron‐Correlated Oxides
Source: Adv Sci (Weinh). 2016 Jan 22;3(5):1500319. doi: 10.1002/advs.201500319 (PMC5066634; doi:10.1002/advs.201500319)
Supplement: Supplementary file 1 — Supplementary [file ADVS-3-0e-s001.pdf]

Copyright WILEY-VCH Verlag GmbH & Co. KGaA, 69469 Weinheim, Germany, 2016.

Supporting Information

for *Adv. Sci.*, DOI: 10.1002/advs. 201500319

Ultrahigh-Power Pseudocapacitors Based on Ordered Porous Heterostructures of Electron-Correlated Oxides

*Xing-You Lang, Bo-Tian Liu, Xiang-Mei Shi, Ying-Qi Li, Zi Wen, and Qing Jiang**

Copyright WILEY-VCH Verlag GmbH & Co. KGaA, 69469 Weinheim, Germany, 2013.

Supporting Information

Ultrahigh-power pseudocapacitors based on ordered porous heterostructures of electron-correlated oxides

Xing-You Lang,[†] *Bo-Tian Liu,*[†] *Xiang-Mei Shi,*[†] *Ying-Qi Li, Zi Wen, Qing Jiang**

Key Laboratory of Automobile Materials (Jilin University), Ministry of Education, and School of Materials Science and Engineering, Jilin University, Changchun 130022, China

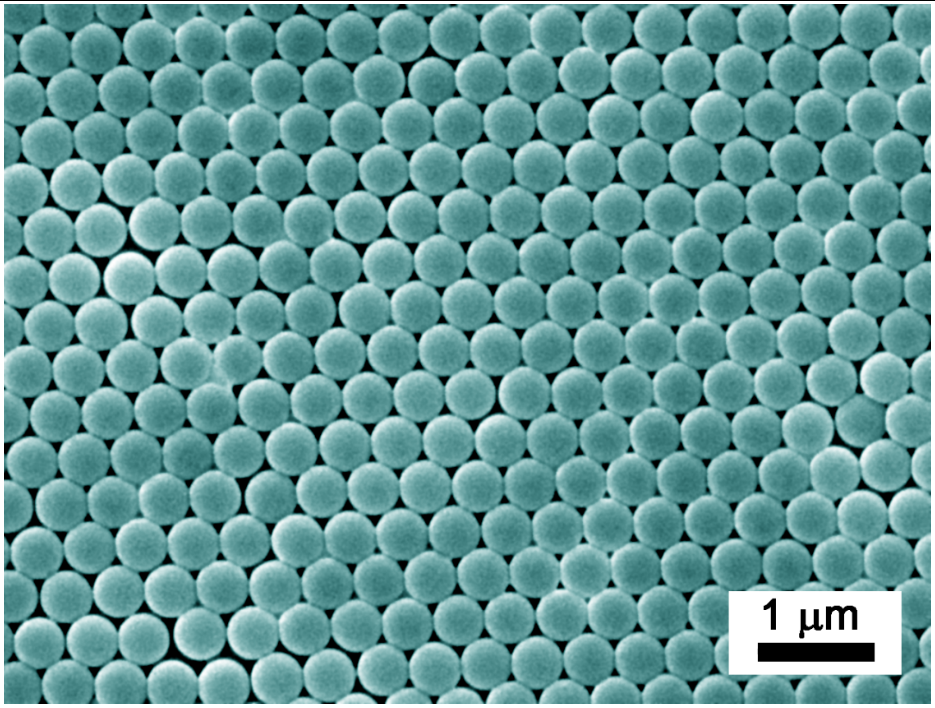

Figure S1. Typical SEM image for NH_4^+-terminated PS spheres self-assembled into opal film on the stainless steel substrate.

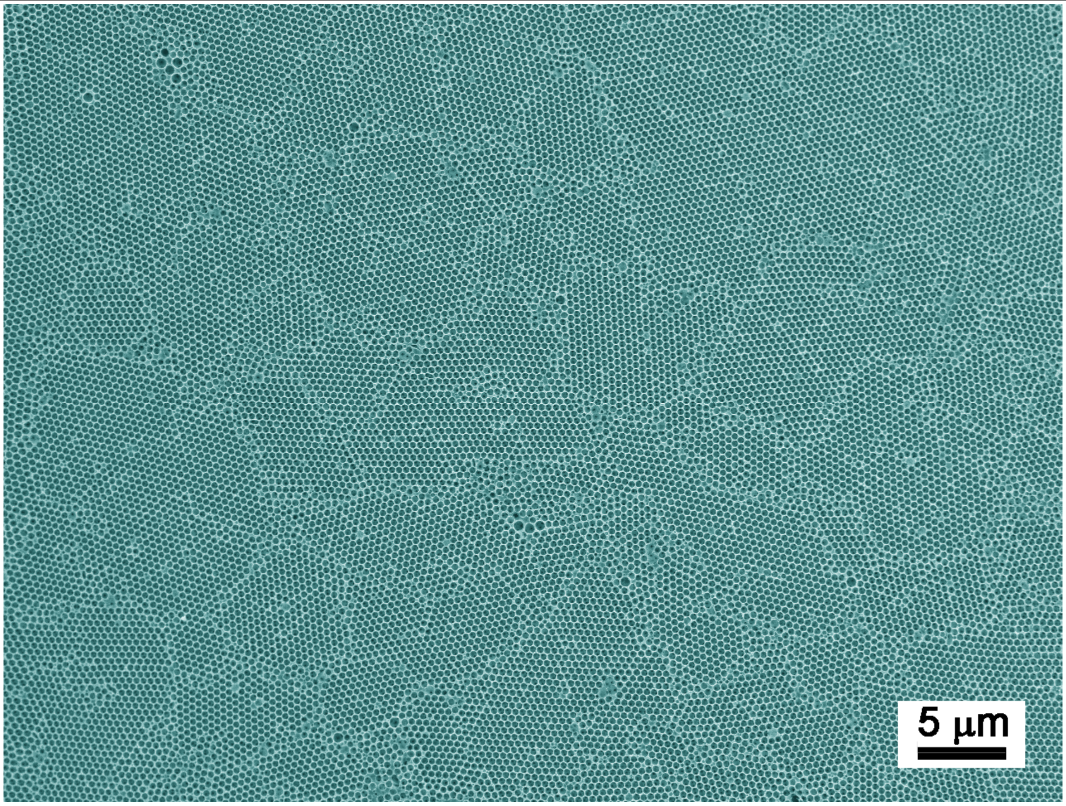

Figure S2. Representative low-magnification SEM image of NP V_2O_3 skeleton supported by current collectors.

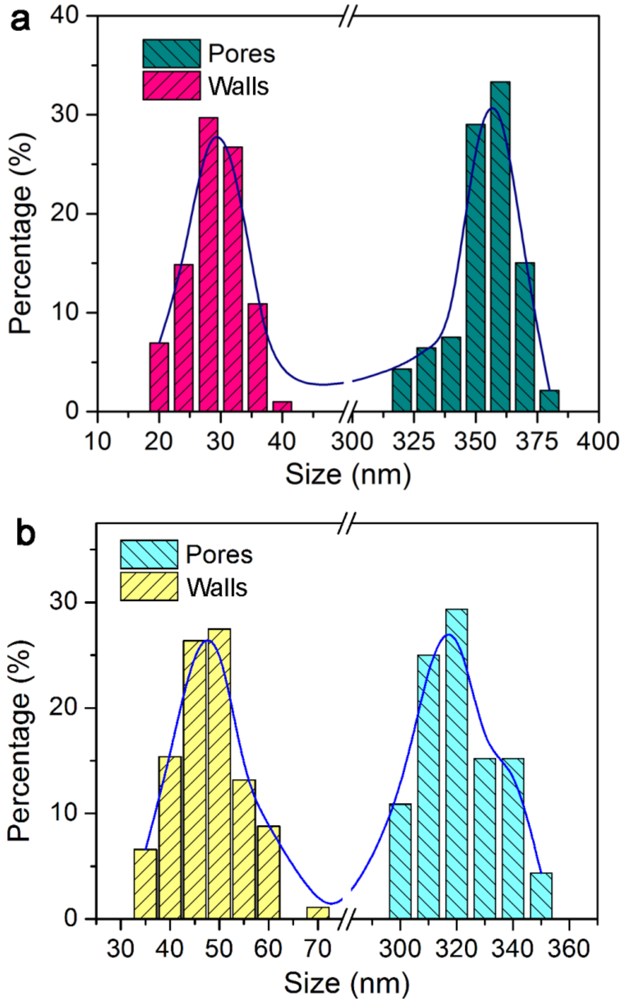

Figure S3. Wall-size and nanopore-size distributions of (**a**) bare NP V_2O_3 skeleton and (**b**) NP V_2O_3/MnO_2 hybrid electrodes.

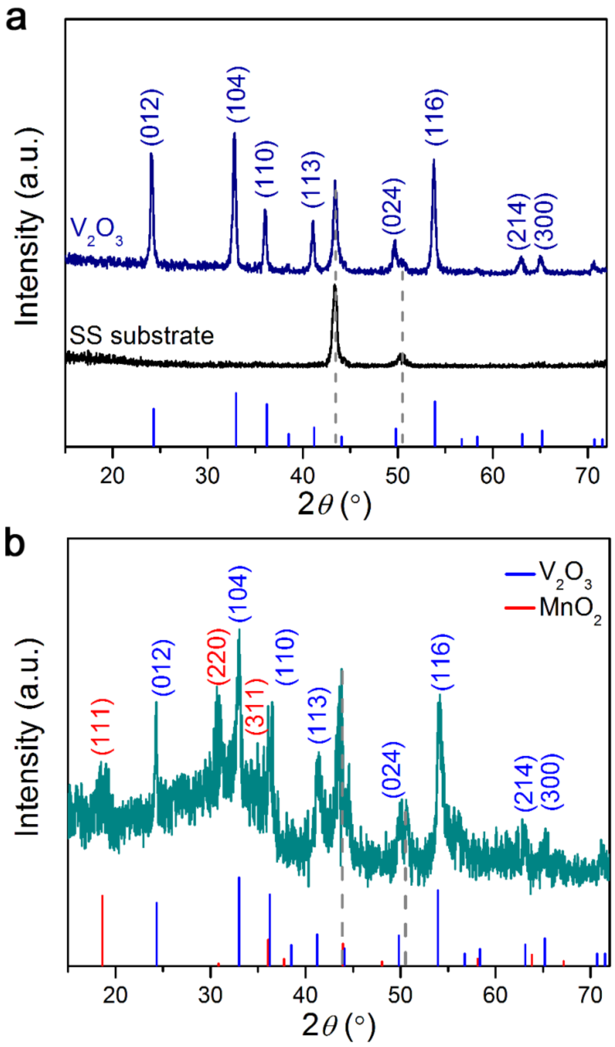

Figure S4. **(a)** XRD pattern of nanoporous V_2O_3 skeleton supported by stainless steel (SS) substrate. The line pattern shows reference card PDF #34-0187 for corundum V_2O_3 according to JCPDS. **(b)** XRD patterns of nanoporous V_2O_3/MnO_2 heterostructure supported by SS substrate. The line patterns are the references for the spinel MnO_2 JCPDS (No. 35-0782) and the corundum V_2O_3 JCPDS (No. 34-0187). The addition peaks at $2\theta = 43.3°$ and $50.6°$ are attributed to SS substrate.

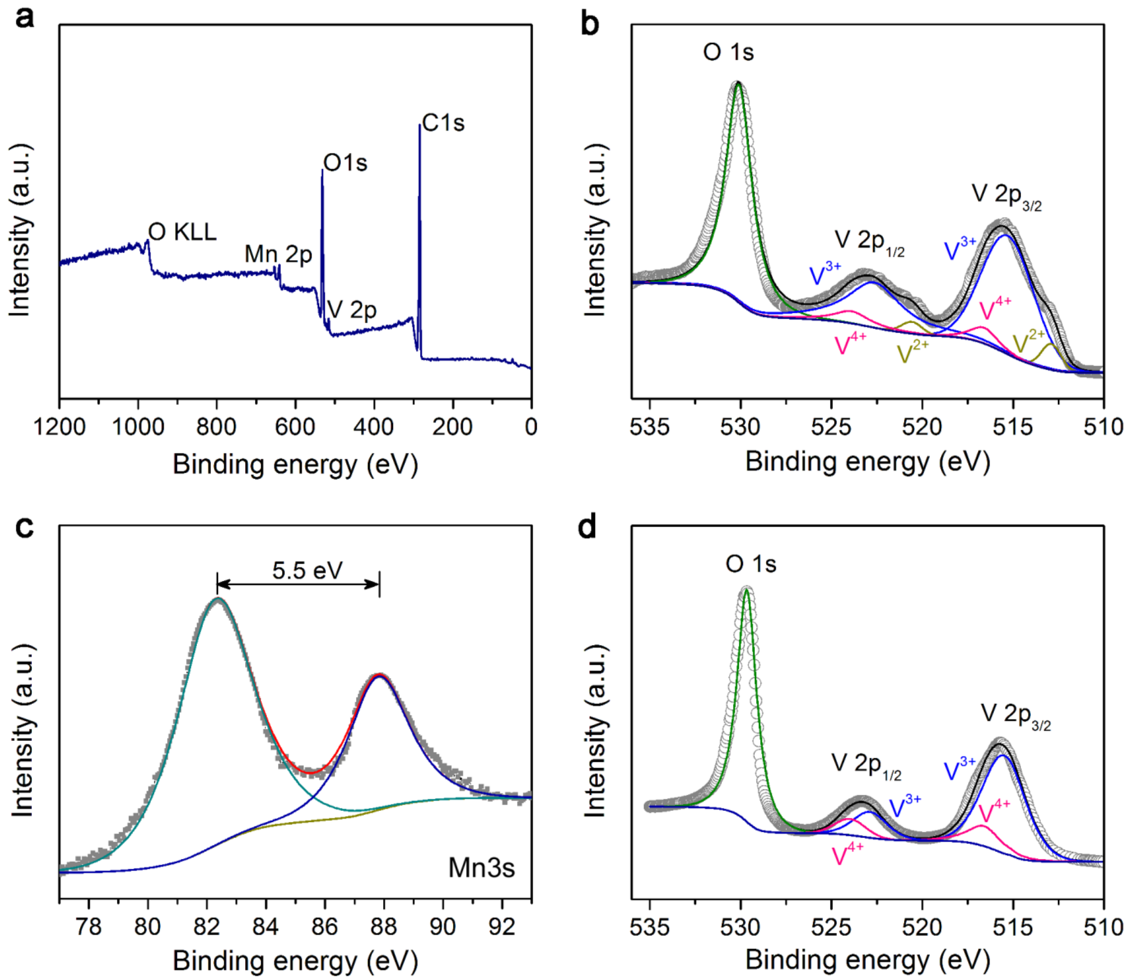

Figure S5. (**a**) Typical XPS survey spectrum for nanoporous V_2O_3/MnO_2 composite electrode. (**b**) High-resolution XPS spectrum of O 1s and V 2p for NP V_2O_3 scaffold. The core binding energies of 516.8 eV, 515.4 eV, 512.9 eV, and 524.1 eV, 522.7 eV, 520.6 eV are attributed to V $2p_{3/2}$ and V $2p_{1/2}$ of V^{4+}, V^{3+} and metallic V^{2+}.[1,2] (**c**) High-resolution XPS spectrum of Mn 3s for the hydrogenated MnO_2. (**d**) High-resolution XPS spectrum and fits for O 1s and V 2p at the V_2O_3/MnO_2 interface. The core binding energies of 515.4 eV, 516.8 eV and 522.7 eV, 524.1 eV corresponding to V $2p_{3/2}$ and V $2p_{1/2}$ are in accordance with those predominant peaks of V^{3+} in V_2O_3, V^{4+} in V-O-Mn bonding. The enhanced intensity of peaks at 516.6 eV and 523.9 eV for V $2p_{3/2}$ and V $2p_{1/2}$ of V^{4+} is due to the formation of V-O-Mn bonding.

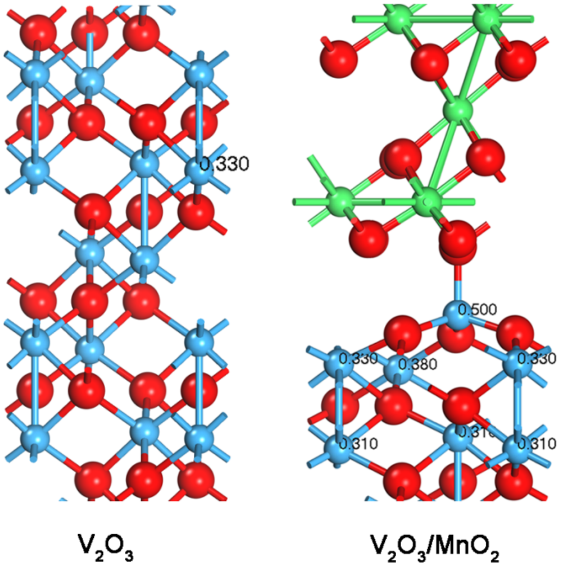

Figure S6. The charge states of the V atoms in the bare V_2O_3 and at the V-O-Mn interface are determined by the Hirshfeld charges. The former (0.330 e) is much lower than the latter (0.500 e) due to the formation of V-O-Mn with remarkable charge transfer.

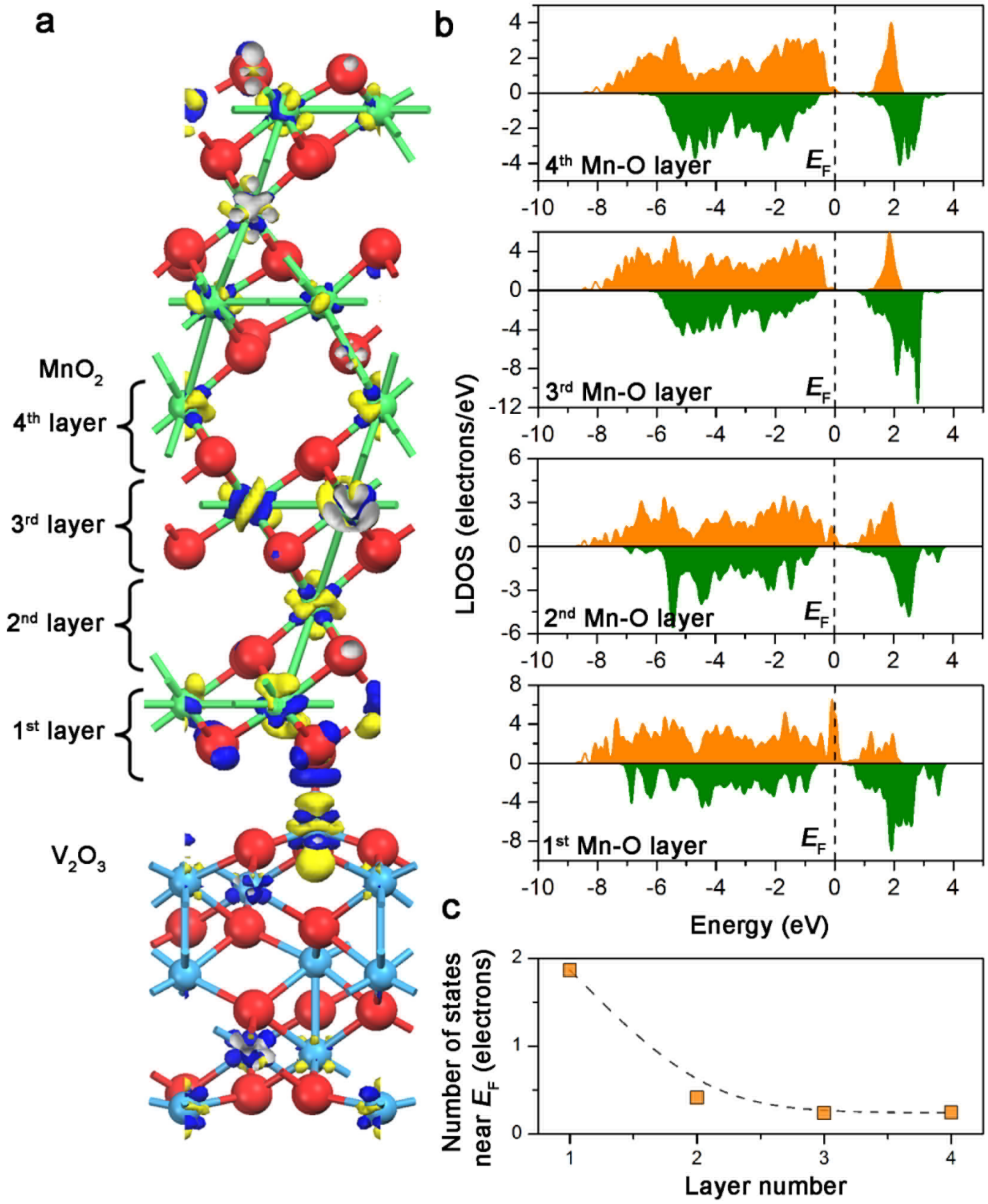

Figure S7. (**a**) Electron-density difference at V_2O_3/MnO_2 interface. Charge transfers from vanadium atom into the O atom with color change from yellow to blue. The isosurface value is 0.06 electrons $Å^{-3}$. (b) Local spin-polarized DOS of four Mn-O layers of MnO_2 (as indicated in **a**) on V_2O_3(0001). (**c**) The number of states of Mn-O layers as a function of layer number away from the V_2O_3(0001) layer. The number of states are obtained for the Mn and O atoms in an energy window of 400 meV centered at the Fermi energy.

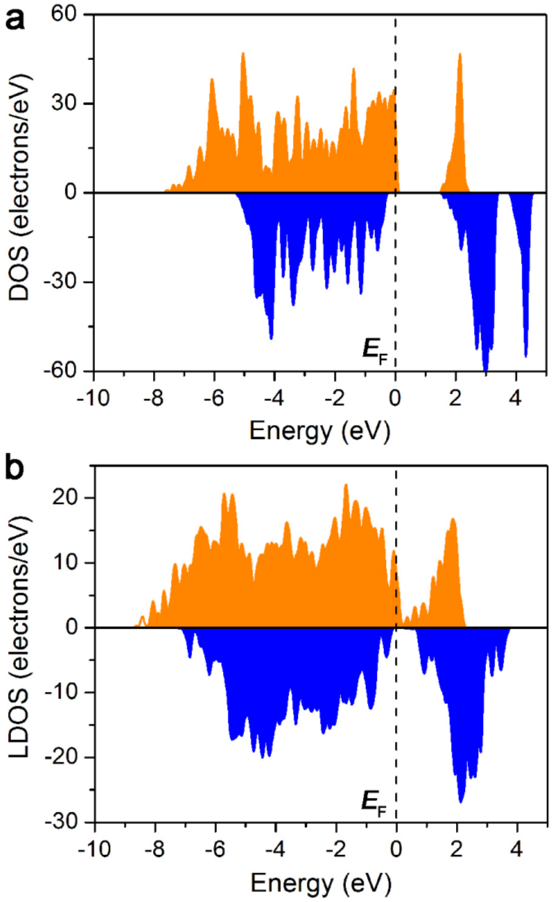

Figure S8. (**a**) Total spin-polarized density of states (DOS) of the spinel MnO_2 bulk. (**b**) Local spin-polarized DOS (LDOS) of spinel MnO_2 layer on V_2O_3(0001) one. Positive DOS is for spin up and negative is for spin down. The dashed line indicates the position of Fermi level E_F.

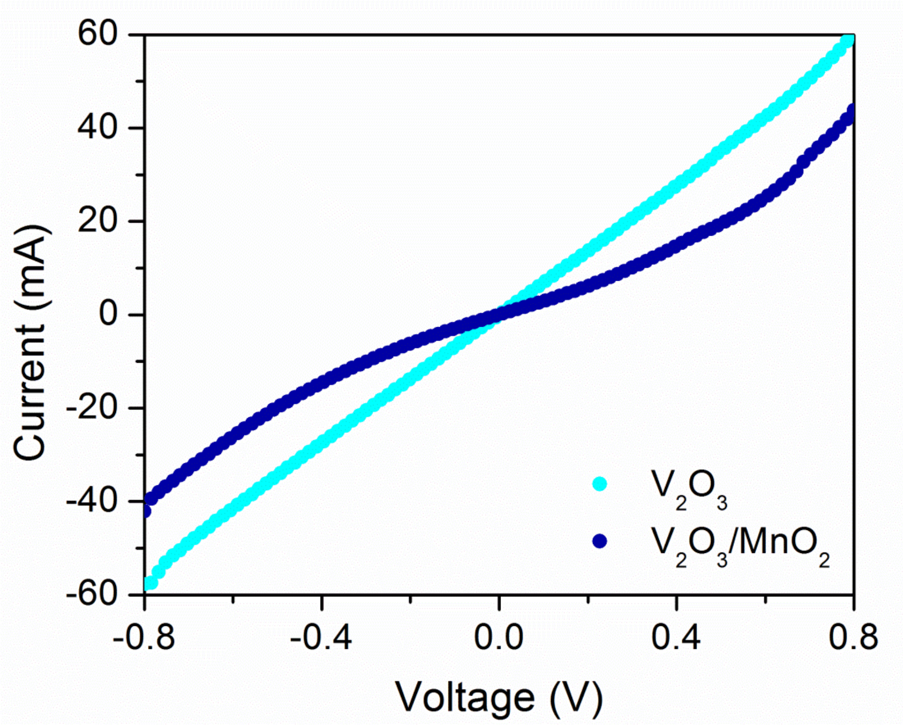

Figure S9. Current-voltage curves for NP V_2O_3 and NP V_2O_3/MnO_2 electrode films.

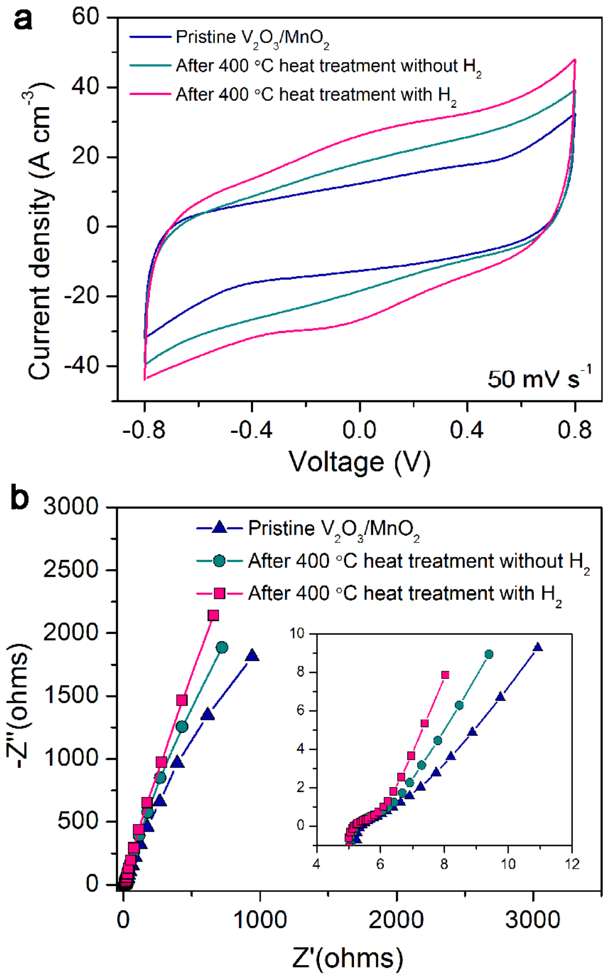

Figure S10. Comparisons of (**a**) CV curves and (**b**) EIS spectra for the pristine V_2O_3/MnO_2 electrodes (plating time, 110 s) and the heat-treated ones in pure Ar and H_2/Ar atmosphere at 400 °C. Inset: A magnification for the high-frequency region.

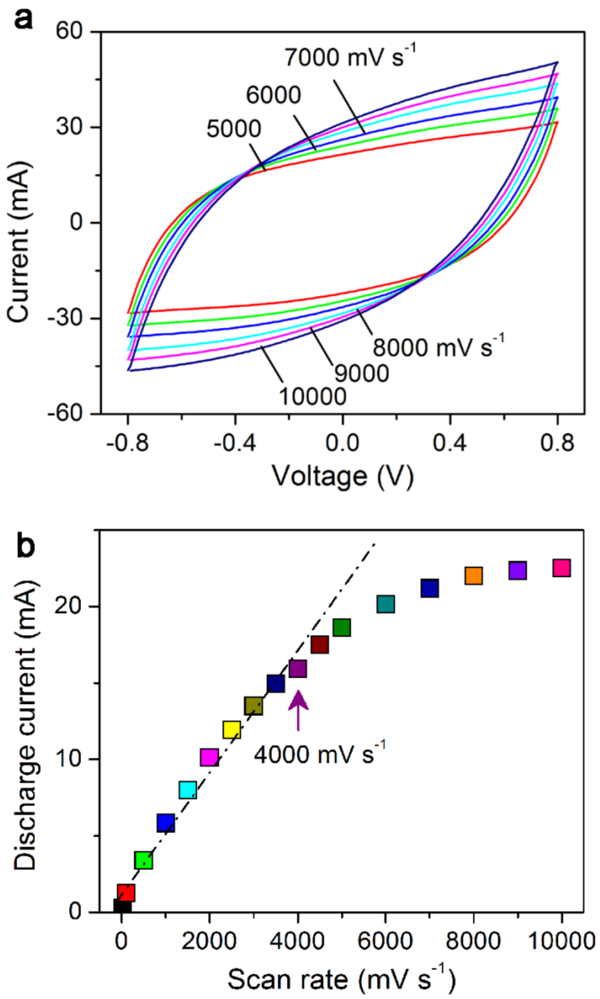

Figure S11. (**a**) CV curves of pseudocapacitors assembled with 47 wt.% MnO_2 loaded NP V_2O_3/MnO_2 electrodes with heat treatment in H_2 air at 400 °C at high scan rates. (**b**) Discharge current at 0.2 V versus scan rate.

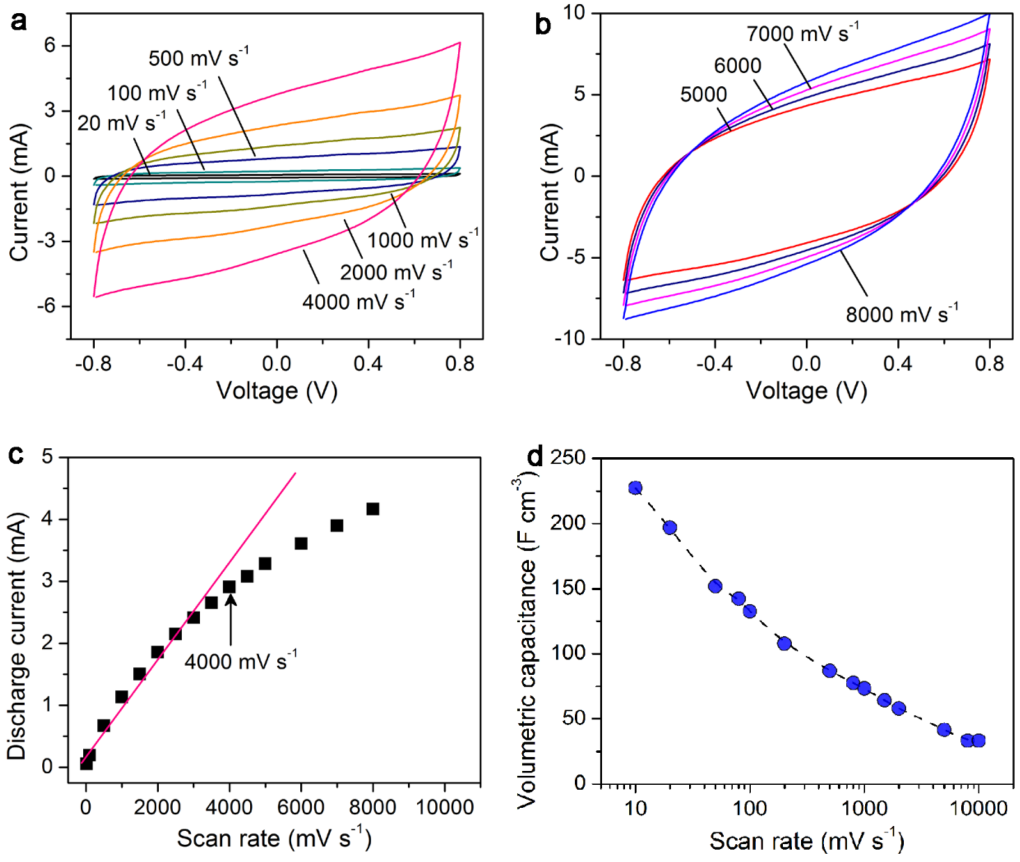

Figure S12. CV curves of pseudocapacitors assembled with NP V_2O_3 electrodes at (**a**) low and (**b**) high scan rates. (**c**) Discharge current at 0.2 V versus scan rate for NP V_2O_3 electrodes. (**d**) Volumetric capacitance of NP V_2O_3 electrodes at various scan rates.

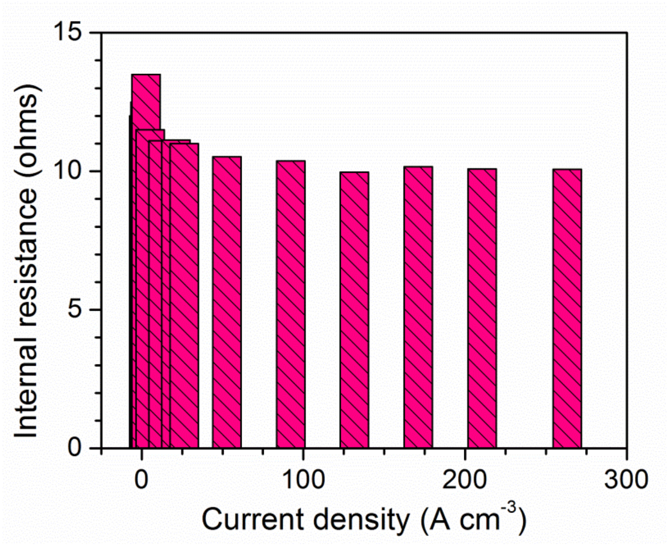

Figure S13. Internal resistances of the pseudocapacitors that are assembled by using NP V_2O_3/MnO_2 electrodes with the 47% MnO_2 loading in 1 M Na_2SO_4 aqueous electrolyte. They are calculated according to the voltage drops at the beginning of each discharge at current density from 1.56 to 312 A cm^{-3}.

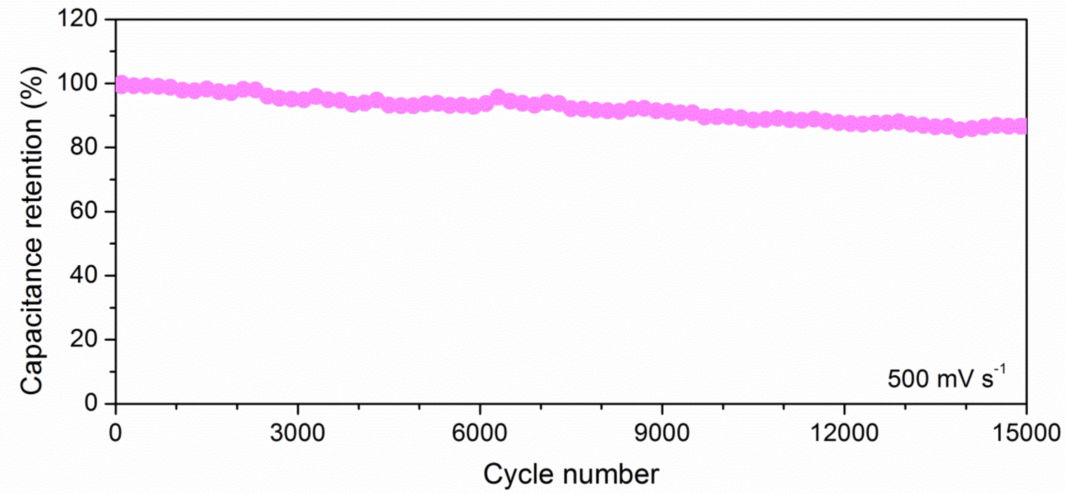

Figure S14. Cycling stability of pseudocapacitor device based on NP V_2O_3/MnO_2 electrodes at the scan rate of 500 mV s^{-1}.

Supplementary Table S1. Geometric features and mass ratio for nanoporous V_2O_3/MnO_2 electrodes at different pulse electrodeposition time.

Sample No.	Electroplating time of MnO_2 (s)	MnO_2 mass ratio (%)
1	0	0
2	10	18.37
3	45	31.03
4	110	47.36

Supplementary references:

[1] E. Hryha, E. Rutqvist, L. Nyborg, *Surf. Interface Anal.* **2012**, *44*, 1022-1025.
[2] G. Silversmit, D. Depla, H. Poelman, G.B. Marin, R. De Gryse, *J. Electron Spectrosc. Relat. Phenom.* **2004**, *135*, 167.
